# Evaluating the accuracy of population-specific versus generic stature estimation regression equations in a South African sample

**DOI:** 10.1007/s00414-024-03340-x

**Published:** 2024-10-02

**Authors:** Mubarak Bidmos, Desiré Brits

**Affiliations:** 1 Department of Basic Medical Sciences, College of Medicine, QU Health, Qatar University, Doha, Qatar; 2 Human Variation and Identification Research Unit (HVIRU), School of Anatomical Sciences, Faculty of Health Sciences, University of the Witwatersrand, Johannesburg, South Africa

**Keywords:** Living stature estimation, Equations validation, Magnetic resonance imaging, Forensic anthropology

## Abstract

Accurate estimates of stature play an important role in the personal identification of unknown decedents, however a drawback in the application of many stature estimation equations is the need for known sex and population, the assignment of which can be challenging. Researchers have formulated equations for stature estimation that are neither population- nor sex-specific and thereof the aim of this study was to assess the applicability of these stature estimation equations proposed by Albanese et al. (2016) (Albanese J, Tuck A, Gomes J, Cardoso HFV (2016) An alternative approach for estimating stature for long bones that is not population- or group-specific. Forensic Sci Int 259:59–68). The maximum length of the femur, condylar malleolar length of the tibia and a combination of these measurements, collected from Magnetic Resonance Imaging Scanograms of adult (20–60 years) White South African males (*n* = 30) and females (*n* = 44) were used to assess the accuracy of the Albanese et al. (Albanese J, Tuck A, Gomes J, Cardoso HFV (2016) An alternative approach for estimating stature for long bones that is not population- or group-specific. Forensic Sci Int 259:59–68). sex-specific and generic stature estimation equations. The stature estimates were compared with measured living stature (LSM), using paired t-tests. Results indicated that the Albanese et al. (Albanese J, Tuck A, Gomes J, Cardoso HFV (2016) An alternative approach for estimating stature for long bones that is not population- or group-specific. Forensic Sci Int 259:59–68). equations underestimated living stature by between 1.2 and 5.0 cm. These underestimations were significantly different between the LSM and the sex-specific estimates for females and the LSM and the generic estimates for males and the tibia for sex-specific equation. All stature estimates however fell in between two standard error of estimates for the sex-specific equations for males and the generic equations for the females. Although, the equations by Albanese et al. (Albanese J, Tuck A, Gomes J, Cardoso HFV (2016) An alternative approach for estimating stature for long bones that is not population- or group-specific. Forensic Sci Int 259:59–68). can be used to estimate stature in White South Africans in certain cases, the use of sex/population-specific equations remains the method of choice.

## Introduction

Documentation of ante-mortem estimates of stature constitutes an important part of the forensic report in addition to other demographic information such as an estimate of age-at-death, sex, and population affinity [1, 2]. Since 1899, when Karl Pearson first published the seminal paper on stature reconstruction, using long bones of the skeleton, forensic and physical anthropologists have shown continued interest in the subject. Consequently, a plethora of studies have been published on stature reconstruction using different bones of the human skeleton with emphasis on the long bones of the upper and lower extremities because of the high correlation of the lengths of these bones with living stature [3].

İşcan and Steyn [3] provided a detailed account of some of the earliest studies on stature estimation from different parts of the world. Trotter and Gleser [4] arguably conducted the largest of such studies in which they presented regression equations for stature estimation using long bones of the upper and lower extremities of Americans of different population groups. They [4] cautioned that regression equations are both population- and sex-specific and these equations should be limited in their application to the populations from which they have been formulated. In addition, they [4] recommended that regression equations should be formulated at different time intervals in order to adapt to the effects of secular trend. As a result, their equations have since been revised [5] and similar population- and sex-specific regression equations have been generated for long bones of limbs in different parts of the world [6–15], including South Africa [16–19].

While it is generally accepted that population specificity of regression equations will increase their precision, the major drawback in their application is the need for prior knowledge of the population group, the assignment of which may be difficult [1]. Consequently, researchers over the years have attempted to formulate equations for stature estimation that are neither population- nor sex-specific [20–22]. Unfortunately, the universal applicability of these equations is unknown as they have not undergone any rigorous testing worldwide [1]. Recently, Albanese et al. [1] developed and proposed generic regression univariate and multivariate equations for stature estimation using long bones of the upper and lower limbs, derived from human remains housed in the Terry Collection. They [1] tested the reliability of the proposed equations in the estimation of living stature on independent samples from the Forensic Anthropology Databank (FDB) and the Lisbon Collection. Findings from the study indicated that the generic equations often outperformed the population-specific Eq. [1]. To date there has not been any independent evaluation of the validity and the reliability of these equations. It is therefore the aim of this study to assess the accuracy of the lower limb generic equations of Albanese et al. [1] in the estimation of living stature using Magnetic Resonance Imaging (MRI) derived data from a sample of contemporary White South Africans.

## Materials and methods

Prior to the commencement of the study, ethical approval (M2111174) was obtained from the Human Research Ethics Committee – Medical of the University of the Witwatersrand, Johannesburg, South Africa. The approval granted the authors of this study access to data that were previously collected as part of two stature reconstruction studies using Magnetic Resonance Imaging (MRI) derived measurements from a contemporary South African population group. Cloete [23] and Loubser et al. [24] collected data including measurements of living stature and MRI derived osteometric measurements from contemporary White South African females (*n* = 44) and males (*n* = 30) respectively. Unfortunately, the sample sizes differed between males and females as some scans had to be removed from the male sample due to technical errors and movement artefacts. Data were collected from invited White South African participants and volunteers who were between 20 and 60 years of age. These ages were particularly selected to ensure that full long bone length had been achieved [25] and that the living statures were not significantly affected by age [26].

In the aforementioned studies by Cloete [23] and Loubser et al. [24], measurement of the living stature (LSM), in the standing position was taken using a stadiometer, followed by a full body MRI scan of each participant in the supine position at Wits-Donald Gordon Medical Centre in Johannesburg, South Africa. The details of how stature related osteometric measurements were taken from the MRI scanograms are provided in a previous study [27]. These measurements included cranial height, height of each vertebra from C2 to S1, physiological (bicondylar) lengths of the femur (FBC), condylar malleolar length of the tibia (TCM) and the talocalcaneal height. The sum total of these measurements is known as the total skeletal height (TSH).

The TCM, as described and illustrated in the previous studies [23, 24, 27], along with the maximum lenght of the femur measure (Fmax) were used in the assessment of the validity of the sex specific and generic regression equations for the estimation of stature by Albanese et al. [1]. To ensure that the dry bone TCM measurement is comparable to the TCM measurement collected from the MRI scan, the tibia was measured according to instructions by Raxter et al. [28] for stature estimation. According to this description, the TCM was measured from the most proximal aspect of the lateral proximal condyle to the tip of the medial malleolus, parallel to the long axis of the bone. Previous research has also shown that there is no significant difference between the dry bone and MRI TCM measurements [29]. Each individual measurement of the Fmax and the TCM as well as a combination of both measurements were substituted into the regression equations that were formulated by Albanese et al. [1] to estimate living stature (ELS) using (i) sex-specific regression equations listed below (numbers 1 to 6) to obtain ELS_AS_ and (ii) generic regression equations (numbers 7 to 9) to obtain ELS_AG_:

### Sex specific equations – females

1. ELS_AS−Fmax_: 0.237 x Fmax + 57.915 (SEE = 3.892).
2. ELS_AS−TCM_: 0.244 x TCM + 73.985 (SEE = 4.425).
3. ELS_AS−FmaxTCM_: 0.165 x Fmax + 0.089 x TCM + 57.360 (SEE = 3.761).

### Sex specific equations – males

4. ELS_AS−Fmax_: 0.250 x Fmax + 55.797 (SEE = 4.781).
5. ELS_AS−TCM_: 0.244 x TCM + 78.999 (SEE = 4.754).
6. ELS_AS−FmaxTCM_: 0.127 x Fmax + 0.131 x TCM + 63.094 (SEE 4.528).

### Generic equations

7. ELS_AG−Fmax_: 0.278 x Fmax + 41.507 (SEE = 4.624).
8. ELS_AG−TCM_: 0.289 x TCM + 59.745 (SEE = 5.068).
9. ELS_AG−FmaxTCM_: 0.183 x Fmax + 0.109 x TCM + 43.790 (SEE = 4.453).

All the measurements and LSM were normally distributed. Mean and standard deviation (SD) as well as the minimum (Min) and maximum (Max) were computed for age, LSM, Fmax, TCM and estimates of living stature obtained from the use of the nine aforementioned sex-specific (ELS_AS_) and generic equations (ELS_AG_) of Albanese et al. [1]. The LSM was then compared to the ELS_AS_ and ELS_AG_. The comparisons were done using paired t-tests which was performed to ascertain whether a statistically significant difference existed between LSM and ELS, with p-values below 0.05 considered as significant. In addition, mean difference (MD), mean absolute deviation (MAD) and the percentage within one and two standard error of estimates (SEE’s) were explored.

The MD is the mean of the difference between the LSM and ELS_AS_ as well as ELS_AG_, while the MAD is the mean of the absolute difference between LSM and ELS_AS_ as well as ELS_AG_. In the calculation of the MD, consideration is given to both the negative and positive differences or errors between LSM and ELS_AS_ or ELS_AG_ in the sample. However, it is the absolute values of these errors that are used in the calculation of the MAD. Therefore, the MAD can be equal to or higher than the MD and it is considered to be a better measure of the overall error because it is the mean of the absolute difference between LSM and ELS_AS_ (or ELS_AG_) [1]. The percentage in range is defined as the number of times that the LSM falls within the range of ELS that was calculated using one and two SEE’s. The number is then expressed as a percentage of the sample size.

In addition, comparison was made between the (i) LSM and estimates of living stature using the recently published population and sex specific regression equations for White South Africans (ELS_B_) by Bidmos et al. [19] and estimates of living statures using Albanese et al. [1] regression equations, and (ii) ELS_B_ and ELS_A_. Analysis of data was performed using SPSS Statistics version 28 and Microsoft Excel version.

## Results

The mean age of females and males were 30.0 and 34.7 years respectively and there was no statistically significant difference between the sexes as shown in Table 1. The mean value for the maximum length of the femur (Fmax), condylar malleolar length of the tibia (TCM) and the measured living stature (LSM) were all statistically significantly larger in males compared to females (Table 1). The mean of the estimated living statures (ELS) using appropriate sex-specific (ELS_AS_) and generic (ELS_AG_) regression equations proposed by Albanese et al. [1] are also summarized in Table 1. Similarly, the ELS in the males were significantly larger than females. Estimates produced using the generic equations (ELS_AG_) were larger than those produced by the sex-specific equation (ELS_AS_) for females, while for males the inverse was noted. These differences were not statistically significant, in either sexes. Except for the stature estimates produced by the FBC (*p* = 0.024) and TCM (*p* = 0.001) in females (Table 1).

**Table 1:** Descriptive statistics

	Females (*n* = 44)				Males (*n* = 30)				t-statistics	*p*-value
	Mean	SD	Min	Max	Mean	SD	Min	Max		
Age (yrs)	30.0	11.6	20.0	60.0	34.7	10.0	22	59	1.8	0.07
Fmax (mm)	445.4	22.5	410.3	492.9	478.3	21.6	436	512	6.3	< 0.0001
TCM (mm)	361.6	21.2	321.1	419.1	392.1	24.5	335	437	5.7	< 0.0001
LSM (cm)	166.4	6.5	152.5	182.6	178.1	6.3	164.8	190.1	7.7	< 0.0001
ELS_AS−Fmax_ (cm)	163.5	5.3	155.1	174.7	175.4	5.4	164.7	183.7	9.4	< 0.0001
ELS_AS−TCM_ (cm)	162.2	5.2	152.3	176.2	174.7	6.0	160.6	185.6	9.5	< 0.0001
ELS_AS−FmaxTCM_ (cm)	163.0	5.5	153.9	174.4	175.2	5.9	162.3	184.8	9.1	< 0.0001
ELS_AG−Fmax_ (cm)	165.3	6.3	155.6	178.5	174.5	6.0	162.4	183.8	6.3	< 0.0001
ELS_AG−TCM_ (cm)	164.2	6.1	152.5	180.9	173.1	7.1	156.3	186.1	5.8	< 0.0001
ELS_AG−FmaxTCM_ (cm)	164.7	6.3	154.2	177.9	174.1	6.5	160.0	184.5	6.2	< 0.0001

Fmax: Maximum length of femur

TCM: Tibia condylomalleolar length

LSM: Living stature measured

ELSAS: Estimated living stature using Albanese et al. [1] sex specific regression equations

ELSAG: Estimated living stature using Albanese et al. [1] generic regression equations

Strong and statistically significant positive correlations were obtained between LSM and all ELS_A_ using the different equations of Albanese et al. [1] (Table 2) for both females and males. The female correlations were stronger than the male correlations. In females, the lowest correlations (*r* = 0.83) were noted for all ELS calculated using the tibia while those calculated using the femur resulted in the lowest correlations (*r* = 0.81) in males. The combined femur and tibia ELS resulted in the strongest correlations for females. In males, sex-specific and generic equations for TCM and that for the sex-specific equations for the combined femur and tibia provided the highest correlation. A comparison of the means between LSM and ELS_AS_ in the female group showed statistically significant differences, except for the equation for Fmax (Table 2). A similar result was obtained in the male group where a statistically significant difference was obtained between LSM and ELS_AG_ except for the equation for Fmax (Table 2). However, no statistically significant differences were observed between the LSM and any of the ELS_AG_ for females, nor the sex-specific equations for the femur and the combined femur and tibia in males (Table 2).

**Table 2:** Correlation coefficient and comparison of means between LSM and ELS_A_ using Albanese et al. [1]. Equations

	Females				Males			
	Correlation coefficient	Coefficient of determination	t-statistics	*p-value	Correlation coefficient	Coefficient of determination	t-statistics	*p-value
ELS_AS−Fmax_ (cm)	0.86	0.74	2.319	**0.023**	0.85	0.72	1.772	0.082
ELS_AS−TCM_ (cm)	0.83	0.69	3.355	**0.001**	0.87	0.76	2.139	**0.037**
ELS_AS−FmaxTCM_ (cm)	0.87	0.76	2.645	**0.010**	0.88	0.77	1.817	0.074
ELS_AG−Fmax_ (cm)	0.86	0.74	0.806	0.423	0.85	0.72	2.258	**0.028**
ELS_AG−TCM_ (cm)	0.83	0.69	1.621	0.109	0.87	0.76	2.892	**0.005**
ELS_AG−FmaxTCM_ (cm)	0.87	0.76	1.258	0.212	0.87	0.76	2.418	**0.019**

LSM: Living stature measured

Fmax: Maximum length of femur

TCM: Tibia condylomalleolar length

ELS_AS_: Estimated living stature using Albanese et al. [1] sex specific regression equation

ELS_AG_: Estimated living stature using Albanese et al. [1] generic regression equation

* paired t-test p-value of comparison between means of LSM and ELS

Similarly, strong and statistically significant positive correlations were also obtained between LSM and all estimates of living stature (ELS_BS_) using the sex-specific regression equations of Bidmos et al. [19] (Table 3) for both sexes. The range of correlation coefficient in females (*r* = 0.83–0.87) and males (*r* = 0.85–0.88) is similar to that obtained for correlations between LSM and ELS_A_. A comparison of the means between LSM and ELS_BS_ in both groups showed no statistically significant differences (Table 3) and shows that the ELS_BS_ using sex specific equations of Bidmos et al. [19] are similar to the LSM.

**Table 3:** Correlation coefficient and comparison of means between LSM and ELS_BS_ using Bidmos et al. [19]. Equations

	Females				Males			
	Correlation coefficient	Coefficient of determination	t-statistics	*p-value	Correlation coefficient	Coefficient of determination	t-statistics	*p-value
ELS_BS−FBC_ (cm)	0.86	0.74	-0.077	0.939	0.85	0.72	-0.133	0.895
ELS_BS−TCM_ (cm)	0.83	0.68	0.078	0.938	0.87	0.76	-0.065	0.948
ELS_BS−FBCTCM_ (cm)	0.87	0.76	0.000	1.000	0.88	0.77	-0.196	0.845

LSM: Living stature measured

FBC: Femur bicondylar length

TCM: Tibia condylomalleolar length

ELS_BS_: Estimated living stature using Bidmos et al. [19] sex specific regression equations

* Paired t-test p-values of comparison between means of LSM and ELS_B_

Table 4 shows the results of a further comparison of the estimates of living stature using sex-specific equations of Albanese et al. [1] and Bidmos et al. [19]. A statistically significant difference exists between ELS_AS_ and ELS_BS_ for all sex-specific equations with the exception of the equations for Fmax in females and males and the combination of Fmax and TCM in males (Table 4). As such, the sex-specific equations of Bidmos et al. [19] generally provide a more accurate estimate of stature compared to the equations of Albanese et al. [1], especially for females.

**Table 4:** Comparison of mean of estimates of living stature using Albanese et al. [1] and Bidmos et al. [19] regression equations

	Females		Males	
	F-statistics	*p-value	F-statistics	*p-value
ELS_AS−Fmax_ - ELS_BS−FBC_ (cm)	2.420	**0.020**	1.810	0.076
ELS_AS−TBC_ - ELS_BS−TBC_ (cm)	3.810	**0.000**	2.220	**0.030**
ELS_AS−FmaxTBC_ - ELS_BS−FBCTBC_ (cm)	2.850	**0.006**	1.766	0.080

Bold values indicate significant difference

ELS_AS_: Estimated living stature using Albanese et al. (1) sex specific regression equation

ELS_BS_: Estimated living stature using Bidmos et al. (19) specific regression equation

In Table 5, it can be seen that the mean difference (MD) between LSM and ELS_AS_ in females ranged between 2.3 cm (for ELS_AS-Fmax_) and 4.2 cm (for ELS_AS-TCM_). The positive MD values indicate that the sex-specific regression equations of Albanese et al. [1] tend to moderately underestimate the LSM of females (Table 5). The values of mean absolute deviation (MAD) between LSM and ELS_AS_ which ranged between 3.5 cm (for ELS_AS-Fmax_) and 4.8 cm (for ELS_AS-TCM_) were higher that the values of the MD (Table 5). This is a further confirmation of the bias of Albanese et al. [1] sex-specific questions in underestimating LSM in females. In addition to MD and MAD, the percentage in range is another measure of the utility of Albanese e*t al.* [1] sex-specific equations and the results are as shown in Table 5. The percent accuracy obtained for females which ranged from 88.6% (for ELS_AS-TCM_) to 90.9% (for ELS_AS-Fmax_ and ELS_AS_FmaxTCM_) is lower compared to that obtained for ELS_AS_ for males (Table 5). In males, a similar magnitude of MD (2.0 cm to 3.4 cm) and MAD (3.4 cm to 4.0 cm) compared to females were obtained with the highest values from sex specific equation for the tibia (Table 5). The positive value of MD and the higher magnitude of MAD also indicates a moderate tendency of the sex-specific regression equations of Albanese et al. [1] to underestimate living stature of males (Table 5). However, the LSM fell within 2SEE’s of ELS_AS_ in 100% of all individuals which indicates a high accuracy of the equations in estimation of living stature in males (Table 5).

**Table 5:** Comparison of living stature measured (LSM) with estimates of stature using Albanese et al. [1] regression equations

Equations	MD	MAD	Accuracy
*Sex-specific equations (Female)*			
ELS_AS−Fmax_ (cm)	2.9	3.8	90.9
ELS_AS−TCM_ (cm)	4.2	4.8	88.6
ELS_AS−FmaxTCM_ (cm)	3.4	4.0	90.9
*Sex-specific equations (Male)*			
ELS_AS−Fmax_ (cm)	2.7	3.5	100.0
ELS_AS−TCM_ (cm)	3.4	4.0	100.0
ELS_AS−FmaxTCM_ (cm)	2.9	3.6	100.0
*Generic equations (Female)*			
ELS_AG−Fmax_ (cm)	1.1	2.9	100.0
ELS_AG−TCM_ (cm)	2.2	3.5	100.0
ELS_AG−FmaxTCM_ (cm)	1.7	3.0	100.0
*Generic equations (Male)*			
ELS_AG−Fmax_ (cm)	3.6	4.2	100.0
ELS_AG−TCM_ (cm)	5.0	5.3	96.7
ELS_AG−FmaxTCM_ (cm)	4.0	4.5	96.7

ELS_AS_: Estimated living stature using Albanese et al. [1] sex-specific regression equation

ELS_AG_: Estimated living stature using Albanese et al. [1] generic regression equation

Table 5 also shows the result of the MD, MAD and percentage in range for the generic equations of Albanese e*t al.* [1]. The MD between LSM and ELS_AG_ in females ranged between 0.3 cm (for ELS_AG-Fmax_) and 2.2 cm (for ELS_AG-TCM_). The MD is lower compared to that obtained for the sex-specific equations for females, indicating a lesser tendency of generic equations to underestimate stature. The observed magnitude of MAD between LSM and ELS_AG_ is also lower than that obtained for the sex- specific equations with a range of 2.7 cm (for ELS_AG-Fmax_) to 3.5 cm (for ELS_AG-TCM_). The magnitude of both MD and MAD is an indication of a lesser tendency of the generic equations of Albanese et al. [1] to underestimate living stature in females. The percentage in range in which the LSM of females fell within two SEE’s of ELS_AG_ using all the equations is 100% (Table 5). Contrary to the observation in the female group, the generic equations underestimate stature with a larger magnitude than the sex-specific equations in males (Table 5). The MD ranged between 2.8 cm (for ELS_AG-Fmax_) and 5.0 cm (for ELS_AG-TCM_) in males. The MAD has a similar magnitude of values compared to MD with a range between 4.1 and 5.3 cm (Table 5). The percentage accuracy in which the LSM lies within 2 SEEs ranged between 96.7% and 100% (Table 5). Compared to the ELS_AS_, the ELS_AG_ produced less accurate LSM in males.

## Discussion

It has been argued that sex- and population-specific stature estimation regression equations produce the most accurate estimates of stature [4, 17, 18]. Unfortunately, the major drawback in the application of these equations is the requirement of sex and population estimates, which in some instances are considered impossible and/or unnecessary [1, 30]. Consequently, a number of researchers have formulated equations for stature estimation that are neither sex- nor population-specific [1, 20–22]. The recent equations proposed by Albanese and colleagues [1] include generic as well as sex-specific equations, which are independent from population affinity. Results regarding the application of these equations have indicated that the generic equations often provide the best estimates of stature. As such, the aim of this study was to explore the use the generic and sex-specific stature estimation regression equations proposed by Albanese et al. [1] for the estimation of stature in White South Africans.

Overall, the results indicated that the Albanese et al. [1] sex-specific equations for the tibia and the combined measurements of the femur and tibia, significantly underestimated female stature and only a maximum of 59.1% of the estimates fell within one standard error of estimate (SEE) increasing to 90.9% for 2SEE’s. This is also echoed in the higher mean absolute deviation (MAD) values associated with the sex-specific equations. Interestingly, only the sex-specific regression equation for the tibia significantly underestimated stature for males and all estimates of stature fell within 70% of one SEE and within a 100% of 2SEE’s. All the male stature estimates produced using the generic equations by Albanese et al. [1] significantly underestimated stature except for the femur with only 43.3–60% of the estimates falling within one SEE and 96.7–100% within 2SEE’s. This is further reflected in the increased MAD values noted when using the generic equations for stature estimation in males. The inverse was noted for females with no significant difference noted between the living stature measured (LSM) and any of the estimates produced using the generic equations. These estimates fell within one SEE in 77.3–86.4% and a 100% within 2SEE’s.

As expected, all the Albanese et al. [1] equations incorrectly estimated stature compared to the population specific equations for White South Africans in which no significant differences were noted between the LSM and ELS_B_ [19]. The underestimations produced by the Albanese et al. [1] equations were only significant for the sex-specific stature estimation equations for females except for the femur as well as the generic stature estimation equations for males except for the femur and the sex-specific equation for the tibia. Albanese et al. [1] also noted that the sex-specific equation for the tibia did not perform well for males from the Forensic Anthropology (FDB) with possible errors in capturing stature being a reason for this. Overall, the largest underestimation generated using the sex-specific equations was for the tibia for both females (4.2 cm) males (3.4 cm). A similar trend was observed for the generic stature estimation equations, however, the magnitude of the underestimation when using the tibia was smaller in the case of females (2.2 cm) compared to the males (5.0 cm). In the past errors in stature estimates produced by equations using the tibia were linked to incorrect measuring of this bone [31]. However, this was not the case in the current study as the tibia measurements used in both studies were collected according to the same definitions. Errors related to the estimates produced by the tibia could however be contributed to the affects of secular trends as research has shown that the tibia is more sensitive to environmental changes compared to the femur [32, 33]. Although Albanese et al. [1] have made every effort to include as much human variation as possible in the developed equations, continuous fluctuating changes in environments are challenging to control for and the resultant secular changes should frequently be explored to ensure applicability of the equations.

Not surprisingly, all stature estimates produced using equations including the maximum length of the femur had the smallest magnitude of underestimation, ranging between 0.3 cm and 2.3 cm for females, and 2.0 cm and 2.8 cm for males. This is to be expected as research has shown that the femur has the strongest correlation to stature [3]. The results of this study indicated that the generic equations produced more accurate estimates of stature for females with 100% of the LSM falling in between 2SEE’s. For males, the sex-specific stature estimation equations produced more accurate results with a 100% of the estimates falling within 2SEE’s. It is expected that the sex-specific equations would generate more accurate results as research has shown that the accuracy of stature estimation equations are sex and population dependent [4, 17, 18]; however, in line with the results by Albanese et al. [1] the generic equations produced some of the most accurate estimates of stature, especially for females.

The more accurate estimates for females using the generic equations could point to the notion that females are less severely affected by environmental factors and as such has more stable statures compare to males [32, 34]. The poorer performance of the sex-specific equations by Albanese et al. [1] for males could also be associated with the difference in height and bone measurements between males in the current study and those individuals in the Terry Collection, used to derive the equations. Unfortunately, the descriptive statistics relating to height and bone measurements were not reported by Albanese et al. [1]. White South Africans (South Africans of European decent) consists of migrants mainly from western European countries including Belgium, the Netherlands, France, Germany, and the United Kingdom. It is generally believed that the admixture of this group of people with indigenous population groups over several years might have changed their genetic make-up [35]. They are, therefore, considered to differ from European and White Americans [35] and could in part explain some of the differences observed. Although the generic equations resulted in larger underestimates for males (2.8–5.0 cm) compared to the females (0.3–2.2 cm) almost all ELS fell within 2SEE’s (96.7–100%).

Establishing generic equations for the estimation of age [36], sex [37] and stature [1] are becoming increasingly important as admixture between populations are becoming progressively more frequent. This increase in gene flow and genetic drift serves to make populations gradually more similar [38] and as such the need for generic equations that produce accurate estimates for any unknown decedent encountered, are required.

In conclusion, results of this study showed that although the sex-specific and generic stature estimation equations proposed by Albanese et al. [1] can be used to estimate stature in White South Africans in certain cases, the use of sex and population-specific equations remains the method of choice. The performance of the equations by Albanese et al. [1] on other South African populations should however also be assessed before adopting these equations into standard forensic anthropological analyses. It should however be kept in mind that the sample size of the current study was small and future studies using larger sample sizes for validation, are encouraged.

## Data Availability

Data available on request, pending the necessary ethical clearances.
