# Correction to: Evaluating the accuracy of population-specific versus generic stature estimation regression equations in a South African sample

**DOI:** 10.1007/s00414-025-03642-8

**Published:** 2025-10-27

**Authors:** Mubarak Bidmos, Desiré Brits

**Affiliations:** 1 Department of Basic Medical Sciences, College of Medicine, QU Health, Qatar University, Doha, Qatar; 2 Human Variation and Identification Research Unit (HVIRU), School of Anatomical Sciences, Faculty of Health Sciences, University of the Witwatersrand, Johannesburg, South Africa

**Correction to: International Journal of Legal Medicine (2025) 139:405–412**

10.1007/s00414-024-03340-x

In the original version, the bicondylar length of the femur was erroneously used in the calculation of the estimated living stature (ELS) using Albanese et al. [1] equations. The maximum length of the femur in the original equations has now been used in the recalculation of ELS and the corrections are as follows:

The Abstract was revised as follows:

The sentence “The physiological length of the femur, condylar malleolar length of the tibia and a combination of these measurements, collected from Magnetic Resonance Imaging Scanograms of adult (20-60 years) White South African males (n = 30) and females (n = 44) were used to assess the accuracy of the Albanese et al. [1] sex-specific and generic stature estimation equations” should be changed to “The maximum length of the femur, physiological length of the femur, condylar malleolar length of the tibia and a combination of these measurements, collected from Magnetic Resonance Imaging Scanograms of adult (20-60 years) White South African males (n = 30) and females (n = 44) were used to assess the accuracy of the Albanese et al. [1] sex-specific and generic stature estimation equations”.

The sentence “Results indicated that the Albanese et al. [1] equations underestimated living stature by between 1.1 and 5.0 cm” should be changed to “Results indicated that the Albanese et al. [1] equations underestimated living stature by between 1.2 and 5.0 cm”

The Materials and methods were revised as follows:

The sentence “Two of these measurements namely the FBC and TCM as described and illustrated in previous studies [23, 24, 27] were used in the assessment of the validity of the sex specific and generic regression equations for the estimation of stature by Albanese *et al*. [1]” should be changed to “The TCM as described and illustrated in previous studies [23, 24, 27] and the maximum length of the femur (Fmax) were used in the assessment of the validity of the sex specific and generic regression equations for the estimation of stature by Albanese *et al*. [1]”.

The sentence “Each individual measurement of the FBC and the TCM as well as a combination of both measurements were substituted into the regression equations that were formulated by Albanese *et al*. [1] to estimate living stature (ELS) using (i) sex-specific regression equations listed below (numbers 1 to 6) to obtain ELS_AS_ and (ii) generic regression equations (numbers 7 to 9) to obtain ELS_AG_:” should be changed to “Each individual measurement of the Fmax and the TCM as well as a combination of both measurements were substituted into the regression equations that were formulated by Albanese *et al*. [1] to estimate living stature (ELS) using (i) sex-specific regression equations listed below (numbers 1 to 6) to obtain ELS_AS_ and (ii) generic regression equations (numbers 7 to 9) to obtain ELS_AG_:”

The sentence “Mean and standard deviation (SD) as well as the minimum (Min) and maximum (Max) were computed for age, LSM, FBC, TCM and estimates of living stature obtained from the use of the nine aforementioned sex-specific (ELS_AS_) and generic equations (ELS_AG_) of Albanese *et al.* [1]” should be changed to “Mean and standard deviation (SD) as well as the minimum (Min) and maximum (Max) were computed for age, LSM, Fmax and TCM and estimates of living stature obtained from the use of the nine aforementioned sex-specific (ELS_AS_) and generic equations (ELS_AG_) of Albanese *et al.* [1].

The Results were revised as follows:

The sentence “The mean value for the physiological (bicondylar) length of the femur (FBC), condylar malleolar length of the tibia (TCM) and the measured living stature (LSM) were all statistically significantly larger in males compared to females (Table 1).” Should be changed to “The mean value for the maximum length of the femur (Fmax) and condylar malleolar length of the tibia (TCM) and the measured living stature (LSM) were all statistically significantly larger in males compared to females (Table 1)”.

The sentence “These differences were not statistically significant, except for the stature estimates produced by the FBC (p=0.024) and TCM (p=0.001) in females (Table 1)” should be changed to “These differences were not statistically significant in either sex (Table 1)”.

Incorrect Table 1

Table 1 Descriptive statistics

**Table:** 

	Females (n=44)				Males (n=30)				t-statistics	p-value
	Mean	SD	Min	Max	Mean	SD	Min	Max		
Age (yrs)	30.0	11.6	20.0	60.0	34.7	10.0	22	59	1.8	0.07
FBC (mm)	445.4	22.5	410.3	492.9	478.3	21.6	436	512	6.3	<0.0001
TCM (mm)	361.6	21.2	321.1	419.1	392.1	24.5	335	437	5.7	<0.0001
LSM (cm)	166.4	6.5	152.5	182.6	178.1	6.3	164.8	190.1	7.7	<0.0001
ELS_AS-FBC_ (cm)	163.5	5.3	155.1	174.7	175.4	5.4	164.7	183.7	9.4	<0.0001
ELS_AS-TCM_ (cm)	162.2	5.2	152.3	176.2	174.7	6.0	160.6	185.6	9.5	<0.0001
ELS_AS-FBCTCM_ (cm)	163.0	5.5	153.9	174.4	175.2	5.9	162.3	184.8	9.1	<0.0001
ELS_AG-FBC_ (cm)	165.3	6.3	155.6	178.5	174.5	6.0	162.4	183.8	6.3	<0.0001
ELS_AG-TCM_ (cm)	164.2	6.1	152.5	180.9	173.1	7.1	156.3	186.1	5.8	<0.0001
ELS_AG-FBCTCM_ (cm)	164.7	6.3	154.2	177.9	174.1	6.5	160.0	184.5	6.2	<0.0001

FBC: Femur bicondylar length

TCM: Tibia condylomalleolar length

LSM: Living stature measured

ELS_AS_: Estimated living stature using Albanese *et al*. [1] sex specific regression equations

ELS_AG_: Estimated living stature using Albanese *et al*. [1] generic regression equations

Corrected Table 1

Table 1 Descriptive statistics

**Table:** 

	Females (n=44)				Males (n=30)				t-statistics	p-value
	Mean	SD	Min	Max	Mean	SD	Min	Max		
Age (yrs)	30.0	11.6	20.0	60.0	34.7	10.0	22	59	1.8	0.07
Fmax (mm)	448.1	23.1	408.4	495.7	481.2	23.1	436	528	6.3	<0.0001
TCM (mm)	361.6	21.2	321.1	419.1	392.1	24.5	335	437	5.7	<0.0001
LSM (cm)	166.4	6.5	152.5	182.6	178.1	6.3	164.8	190.1	7.7	<0.0001
ELS_AS-Fmax_ (cm)	164.1	5.5	154.8	175.4	176.1	5.8	164.7	187.7	9.4	<0.0001
ELS_AS-TCM_ (cm)	162.2	5.2	152.3	176.2	174.7	6.0	160.6	185.6	9.5	<0.0001
ELS_AS-FmaxTCM_ (cm)	163.5	5.6	153.4	175.2	175.6	6.0	162.3	185.6	9.1	<0.0001
ELS_AG-Fmax_ (cm)	166.1	6.4	155.1	179.3	175.3	6.4	162.6	188.2	6.3	<0.0001
ELS_AG-TCM_ (cm)	164.2	6.1	152.5	180.9	173.1	7.1	156.3	186.1	5.8	<0.0001
ELS_AG-FmaxTCM_ (cm)	164.2	6.4	153.6	178.8	174.6	6.7	160.0	185.8	6.2	<0.0001

Fmax: Maximum length of femur

TCM: Tibia condylomalleolar length

LSM: Living stature measured

ELS_AS_: Estimated living stature using Albanese *et al*. [1] sex specific regression equations

ELS_AG_: Estimated living stature using Albanese *et al*. [1] generic regression equations

The sentence “In females, the lowest correlations (r=0.83) were noted for all ELS calculated using the tibia while those calculated using the femur resulted in the lowest correlations (r=0.85) in males” should be changed to “In females, the lowest correlations (r=0.83) were noted for all ELS calculated using the tibia while those calculated using the femur resulted in the lowest correlations (r=0.81) in males”.

The sentence “The combined femur and tibia ELS resulted in the strongest correlations for females and for the sex-specific ELS in males” should be changed to “The combined femur and tibia ELS resulted in the strongest correlations for females. In males, sex-specific and generic equations for TCM and that for the sex-specific equations for the combined femur and tibia provided the highest correlation”.

The sentence “A comparison of the means between LSM and ELS_AS_ in the female group showed statistically significant differences (Table 2)’ should be changed to “A comparison of the means between LSM and ELS_AS_ in the female group showed statistically significant differences except for the equation for Fmax (Table 2)”.

The sentence “A similar result was obtained for ELS_AS-TCM_ and all the ELS_AG_ in the male group (Table 2)” should be changed to “A similar result was obtained in the male group where a statistically significant difference was obtained between LSM and ELS_AG_ except for the equation for Fmax (Table 2)”.

Incorrect Table 2

Table 2 Correlation coefficient and comparison of means between LSM and ELS_A_ using Albanese *et al*. [1] equations

**Table:** 

	Females				Males			
	Correlation coefficient	Coefficient of determination	t-statistics	*p-value	Correlation coefficient	Coefficient of determination	t-statistics	*p-value
ELS_AS-FBC_ (cm)	0.86	0.74	2.319	**0.023**	0.85	0.72	1.772	0.082
ELS_AS-TCM_ (cm)	0.83	0.69	3.355	**0.001**	0.87	0.76	2.139	**0.037**
ELS_AS-FBCTCM_ (cm)	0.87	0.76	2.645	**0.010**	0.88	0.77	1.817	0.074
ELS_AG-FBC_ (cm)	0.86	0.74	0.806	0.423	0.85	0.72	2.258	**0.028**
ELS_AG-TCM_ (cm)	0.83	0.69	1.621	0.109	0.87	0.76	2.892	**0.005**
ELS_AG-FBCTCM_ (cm)	0.87	0.76	1.258	0.212	0.87	0.76	2.418	**0.019**

LSM: Living stature measured

FBC: Femur bicondylar length

TCM: Tibia condylomalleolar length

ELS_AS_: Estimated living stature using Albanese et al. [1] sex specific regression equation

ELS_AG_: Estimated living stature using Albanese et al. [1] generic regression equation

* paired t-test p-value of comparison between means of LSM and ELS

Corrected Table 2

Table 2 Correlation coefficient and comparison of means between LSM and ELS_A_ using Albanese *et al*. [1] equations

**Table:** 

	Females				Males			
	Correlation coefficient	Coefficient of determination	t-statistics	*p-value	Correlation coefficient	Coefficient of determination	t-statistics	*p-value
ELS_AS-Fmax_ (cm)	0.86	0.74	1.792	0.077	0.81	0.66	1.279	0.206
ELS_AS-TCM_ (cm)	0.83	0.69	3.347	**0.001**	0.87	0.76	2.141	**0.037**
ELS_AS-FmaxTCM_ (cm)	0.87	0.76	2.242	**0.003**	0.87	0.76	1.574	0.121
ELS_AG-Fmax_ (cm)	0.86	0.74	0.218	0.828	0.81	0.66	1.708	0.093
ELS_AG-TCM_ (cm)	0.83	0.69	1.637	0.105	0.87	0.76	2.885	**0.006**
ELS_AG-FmaxTCM_ (cm)	0.87	0.76	0.873	0.385	0.86	0.74	2.084	**0.042**

LSM: Living stature measured

Fmax: Maximum length of femur

TCM: Tibia condylomalleolar length

ELS_AS_: Estimated living stature using Albanese et al. [1] sex specific regression equation

ELS_AG_: Estimated living stature using Albanese et al. [1] generic regression equation

* paired t-test p-value of comparison between means of LSM and ELS

The sentence “A statistically significant difference exists between ELS_AS_ and ELS_BS_ for all sex-specific equations with the exception of the equations for FBC and the combination of FBC and TCM in males (Table 4) should be changed to “A statistically significant difference exists between ELS_AS_ and ELS_BS_ for all sex-specific equations with the exception of the equations for Fmax in females and males, and the combination of Fmax and TCM in males (Table 4)”.

Incorrect Table 4

Table 4 Comparison of mean of estimates of living stature using Albanese et al. [1] and Bidmos et al. [19] regression equations

**Table:** 

	Females		Males	
	F-statistics	*p-value	F-statistics	*p-value
ELS_AS-FBC_ - ELS_BS-FBC_ (cm)	2.420	**0.020**	1.810	0.076
ELS_AS-TBC_ - ELS_BS-TBC_ (cm)	3.810	**0.000**	2.220	**0.030**
ELS_AS-FBCTBC_ - ELS_BS-FBCTBC_ (cm)	2.850	**0.006**	1.766	0.080

Bold values indicate significant difference

ELS_AS_: Estimated living stature using Albanese et al. (1) sex specific regression equation

ELS_BS_: Estimated living stature using Bidmos et al. (19) specific regression equation

* paired t-test p-value of comparison between means of ELS_BS_ and ELS_AS_

Corrected Table 4

Table 4 Comparison of mean of estimates of living stature using Albanese et al. [1] and Bidmos et al. [19] regression equations

**Table:** 

	Females		Males	
	F-statistics	*p-value	F-statistics	*p-value
ELS_AS-Fmax_ - ELS_BS-FBC_ (cm)	1.859	0.066	1.381	0.172
ELS_AS-TBC_ - ELS_BS-TBC_ (cm)	3.810	**0.000**	2.220	**0.030**
ELS_AS-FmaxTBC_ - ELS_BS-FBC TBC_ (cm)	2.407	**0.018**	1.480	0.144

Bold values indicate significant difference

FBC: Femur bicondylar length

ELS_AS_: Estimated living stature using Albanese et al. (1) sex specific regression equation

ELS_BS_: Estimated living stature using Bidmos et al. (19) specific regression equation

* paired t-test p-value of comparison between means of ELS_BS_ and ELS_AS_

The sentence “In Table 5, it can be seen that the mean difference (MD) between LSM and ES in females ranged between 2.9 cm (for ELS_AS-FBC_) and 4.2 cm (for ELS_AS-TCM_)” should be changed to “In Table 5, it can be seen that the mean difference (MD) between LSM and ELS_AS_ in females ranged between 2.3 cm (foLSAr ELS_AS-Fmax_) and 4.2 cm (for ELS_AS-TCM_)”

The sentence “The values of mean absolute deviation (MAD) between LSM and ELS_AS_ which ranged between 3.8 cm (for ELS_AS-FBC_) and 4.8 cm (for ELS_AS-TCM_) were higher that the values of the MD (Table 5)” should be changed to “The values of mean absolute deviation (MAD) between LSM and ELS_AS_ which ranged between 3.5 cm (for ELS_AS-Fmax_) and 4.8 cm (for ELS_AS-TCM_) were higher that the values of the MD (Table 5)”.

The sentence “The percent accuracy obtained for females which ranged from 88.6% (for ELS_AS-TCM_) to 90.9% (for ELS_AS-FBC_ and ELS_AS_FBCTCM_) is lower compared to that obtained for ELS_AS_ for males (Table 5)” should be changed to “The percent accuracy obtained for females which ranged from 88.6% (for ELS_AS-TCM_) to 90.9% (for ELS_AS-Fmax_ and ELS_AS_FmaxTCM_) is lower compared to that obtained for ELS_AS_ for males (Table 5)”.

The sentence “In males, a similar magnitude of MD (2.7 cm to 3.4 cm) and MAD (3.5 cm to 4.0 cm) compared to females were obtained with the highest values from sex specific equation for the tibia (Table 5)” should be changed to “In males, a similar magnitude of MD (2.0 cm to 3.4 cm) and MAD (3.4 cm to 4.0 cm) compared to females were obtained with the highest values from sex specific equation for the tibia (Table 5)”

Incorrect Table 5

Table 5 Comparison of living stature measured (LSM) with estimates of stature using Albanese et al. [1] regression equations

**Table:** 

Equations	MD	MAD	Accuracy
Sex-specific equations (Female)			
ELS_AS-FBC_ (cm)	2.9	3.8	90.9
ELS_AS-TCM_ (cm)	4.2	4.8	88.6
ELS_AS-FBCTCM_ (cm)	3.4	4.0	90.9
Sex-specific equations (Male)			
ELS_AS-FBC_ (cm)	2.7	3.5	100.0
ELS_AS-TCM_ (cm)	3.4	4.0	100.0
ELS_AS-FBCTCM_ (cm)	2.9	3.6	100.0
Generic equations (Female)			
ELS_AG-FBC_ (cm)	1.1	2.9	100.0
ELS_AG-TCM_ (cm)	2.2	3.5	100.0
ELS_AG-FBCTCM_ (cm)	1.7	3.0	100.0
Generic equations (Male)			
ELS_AG-FBC_ (cm)	3.6	4.2	100.0
ELS_AG-TCM_ (cm)	5.0	5.3	96.7
ELS_AG-FBCTCM_ (cm)	4.0	4.5	96.7

ELS_AS_: Estimated living stature using Albanese et al. [1] sex-specific regression equation

ELS_AG_: Estimated living stature using Albanese et al. [1] generic regression equation

Corrected Table 5

Table 5 Comparison of living stature measured (LSM) with estimates of stature using Albanese et al. [1] regression equations

**Table:** 

Equations	MD	MAD	Accuracy
Sex-specific equations (Female)			
ELS_AS-Fmax_ (cm)	2.3	3.5	90.9
ELS_AS-TCM_ (cm)	4.2	4.8	88.6
ELS_AS-FmaxTCM_ (cm)	2.9	3.7	90.9
Sex-specific equations (Male)			
ELS_AS-Fmax_ (cm)	2.0	3.5	100.0
ELS_AS-TCM_ (cm)	3.4	4.0	100.0
ELS_AS-FmaxTCM_ (cm)	2.5	3.4	100.0
Generic equations (Female)			
ELS_AG-Fmax_ (cm)	0.3	2.7	100.0
ELS_AG-TCM_ (cm)	2.2	3.5	100.0
ELS_AG-FmaxTCM_ (cm)	1.2	2.9	100.0
Generic equations (Male)			
ELS_AG-Fmax_ (cm)	2.8	4.1	100.0
ELS_AG-TCM_ (cm)	5.0	5.3	96.7
ELS_AG-FmaxTCM_ (cm)	3.5	4.3	96.7

ELS_AS_: Estimated living stature using Albanese et al. [1] sex-specific regression equation

ELS_AG_: Estimated living stature using Albanese et al. [1] generic regression equation

The sentence “The MD between LSM and ELS_AG_ in females ranged between 1.1 cm (for ELS_AG-FBC_) and 2.2 cm (for ELS_AG-TCM_)” should be changed to “The MD between LSM and ELS_AG_ in females ranged between 0.3 cm (for ELS_AG-Fmax_) and 2.2 cm (for ELS_AG-TCM_)”.

The sentence “The observed magnitude of MAD between LSM and ELS_AG_ is also lower than that obtained for the sex- specific equations with a range of 2.9 cm (for ELS_AG-FBC_) to 3.5 cm (for ELS_AG-TCM_)” should be changed to “The observed magnitude of MAD between LSM and ELS_AG_ is also lower than that obtained for the sex- specific equations with a range of 2.7 cm (for ELS_AG-Fmax_) to 3.5 cm (for ELS_AG-TCM_)”.

The sentence “The MD ranged between 3.6 cm (for ELS_AG-FBC_) and 5.0 cm (for ELS_AG-TCM_) in males. The MAD has a similar magnitude of values compared to MD with a range between 4.2 and 5.3 cm (Table 5)” should be changed to “The MD ranged between 2.8 cm (for ELS_AG-Fmax_) and 5.0 cm (for ELS_AG-TCM_) in males”.

The sentence “The MAD has a similar magnitude of values compared to MD with a range between 4.2 and 5.3 cm (Table 5)” should be changed to “The MAD has a similar magnitude of values compared to MD with a range between 4.1 and 5.3 cm (Table 5)”.

The Discussion was revised as follows:

The sentence “Overall, the results indicated that the Albanese et al. [1]. sex-specific equations for the femur, tibia and the combined measurements of the femur and tibia, significantly underestimated female stature and only a maximum of 59.1% of the estimates fell within one standard error of estimate (SEE) increasing to 90.9% for 2SEE’s”. should be changed to “Overall, the results indicated that the Albanese et al. [1]. sex-specific equations for the tibia and the combined measurements of the femur and tibia, significantly underestimated female stature and only a maximum of 59.1% of the estimates fell within one standard error of estimate (SEE) increasing to 90.9% for 2SEE’s”.

The sentence “All the male stature estimates produced using the generic equations by Albanese et al. [1]. significantly underestimated stature with only 43.3–60% of the estimates falling within one SEE and 96.7–100% within 2SEE’s” should be changed to “All the male stature estimates produced using the generic equations by Albanese et al. [1]. significantly underestimated stature except for the femur with only 43.3–60% of the estimates falling within one SEE and 96.7–100% within 2SEE’s”.

The sentence “The underestimations produced by the Albanese et al. [1]. equations were only significant for the sex-specific stature estimation equations for females as well as the generic stature estimation equations for males and the sex-specific equation for the tibia” should be changed to “The underestimations produced by the Albanese et al. [1]. equations were only significant for the sex-specific stature estimation equations for females except for the femur as well as the generic stature estimation equations for males except for the femur and the sex-specific equation for the tibia”

The sentence “Not surprisingly, all stature estimates produced using equations including the length of the femur had the smallest magnitude of underestimation, ranging between 1.1 cm and 2.9 cm for females, and 2.7 cm and 3.6 cm for males” should be changed to “Not surprisingly, all stature estimates produced using equations including the maximum length of the femur had the smallest magnitude of underestimation, ranging between 0.3 cm and 2.3 cm for females, and 2.0 cm and 2.8 cm for males”.

The sentence “Although the generic equations resulted in larger underestimates for males (3.6-5.0 cm) compared to the females (1.1-2.2 cm) almost all ELS fell within 2SEE’s (96.7-100.7%)” should be changed to “Although the generic equations resulted in larger underestimates for males (2.8-5.0 cm) compared to the females (0.3-2.2 cm) almost all ELS fell within 2SEE’s (96.7-100%)”.

The original article has been corrected.

